# Molecular, Biochemical and Ultrastructural Changes Induced by Pb Toxicity in Seedlings of *Theobroma cacao* L.

**DOI:** 10.1371/journal.pone.0129696

**Published:** 2015-07-06

**Authors:** Graciele Santos Monteiro Reis, Alex-Alan Furtado de Almeida, Nicolle Moreira de Almeida, Andressa Vieira de Castro, Pedro Antonio Oliveira Mangabeira, Carlos Priminho Pirovani

**Affiliations:** Department of Biological Sciences, State University of Santa Cruz, Campus Soane Nazaré de Andrade, Rodovia Jorge Amado, km 16, 45662–900, Ilhéus, BA, Brazil; Kobe University, JAPAN

## Abstract

Pb is a metal which is highly toxic to plants and animals, including humans. High concentrations of Pb have been observed in beans of *T*. *cacao*, as well as in its products. In this work, we evaluated the molecular, biochemical, and ultrastructural alterations in mature leaves and primary roots of seedlings of two progenies of *T*. *cacao*, obtained from seed germination in different concentrations of Pb (0, 0.05, 0.1, 0.2, 0.4, 0.8 g L*^-1^*), in the form of Pb(NO_3_)_2_. The progenies resulted from self-fertilization of *Catongo* and a cross of *CCN-10 x SCA-6*. The Pb, supplied via seminal, caused alterations in the ultrastructures of the mesophyll cells and in the amount of starch grains in the chloroplasts. The dosage of substances reactive to thiobarbituric acid showed that Pb induced lipid peroxidation. The activity of guaiacol peroxidases and the expression of genes associated to synthetase of phytochelatin, *SODcyt* and *PER* increased in response to Pb. In addition, there was alteration in the expression of stress-related proteins. The progeny of *CCN-10 x SCA-6* was more tolerant to Pb stress when compared to *Catongo*, since: (i) it accumulated more Pb in the roots, preventing its translocation to the shoot; (ii) it presented higher activity of peroxidases in the roots, which are enzymes involved in the elimination of excess of reactive oxygen species; and (iii) increased expression of the gene in the phytochelatin biosynthesis route. The results of the proteomic analysis were of paramount importance to differentiate the defense mechanisms used by both progenies of *T*. *cacao*.

## Introduction

*Theobroma cacao* L. is a perennial woody species, preferably allogamous [1,2] of great economic importance. Its fermented and dried seeds (beans) are the main raw material of chocolate. The approximate annual production is four million tons worldwide [2]. High concentrations of Pb have been detected in both *T*. *cacao* and its products [3]. Contamination of *T*. *cacao* by Pb may be attributed to several sources, including: extensive processing of mining, foundry, automotive paints, cellulose and paper, and explosives [4,5]. In addition, the increasing use of phosphate fertilizers has been one of the main means of soil contamination by heavy metals, among which Pb stands out [6,7]. This metallic element is highly toxic to plants, animals, and humans [8].

Excess of Pb in plants can alter a series of biological mechanisms. It can affect seed germination [9], cause reduction in growth, promote leaf chlorosis and darkening of the root system [10], reduce stomatal conductance and size of the stomata [11], alter the activity of enzymes [12], inhibit photosynthesis due to disturbances in the electron transfer reaction [13–15], reduce respiratory rate [16], interfere on mineral nutrition and water balance, promote changes in hormonal status and affect the structure and permeability of membranes [17–19].

Plants absorb and accumulate Pb in roots, stems, leaves, root nodules, and seeds, and this increase depends on the enhancement of the exogenous levels of Pb [20]. A large part of the Pb absorbed by plants accumulates in the roots, and a small fraction is translocated to the shoots [21,22]. The retention of Pb in roots is based on sites of connections of exchangeable ions and on extracellular precipitation, mostly in the form of Pb carbonates, both these mechanisms occuring in the cell wall [22–24]. However, Pb does not always penetrate the root endodermis and enter the stele. Thus, the endoderm acts as a barrier to the absorption of Pb into the stele and its transport to the shoots [25,26].

Tolerance and/or resistance of plants to metal stress may be associated to one or more mechanisms, such as: excretion of chelating compounds that reduce the availability of the metal in soil or in water [4,27]; the exclusion of the metal by means of selective absorption of elements; metal retention in the roots, avoiding its translocation to the shoots [28]; chelation or sequestration of heavy metals by binders, biotransformation, compartmentalization, and cell repair mechanisms [29]; the development of metal-tolerant enzymes [12]; increased production of intracellular compounds bound to the metal [4]; immobilization of the metal in the cell wall [26,30]; the cellular homeostatic mechanisms to regulate the concentration of metal ions inside the cell [13]; the induction of heat shock proteins [31]; and the release of phenols from roots.[32]. Pb can increase the activity of enzymes involved in oxidative stress and in the expression of respective genes, such as glutathione reductase, glutathione S-transferase, ascorbate peroxidase, and superoxide dismutase [33–36]. The oxidative stress, induced by Pb, can generate large amounts of reactive oxygen species (ROSs) [19,37], such as superoxide, hydroxyl, hydrogen peroxide, and singlet oxygen, which are involved in all areas of aerobic metabolism and usually are also associated to the damage to membranes and the reconstruction of lipid peroxidation and chromosomal modifications [38]. In addition, the study of protein expression induced by heavy metal stress has been widely reported in the literature, such as for *Helianthus annuus* exposed to Pb [39] and for *Populus nigra* exposed to Cd [40].

In the present work, different responses were found in progenies of *T*. *cacao* when exposed to high concentrations of Pb. Ultrastructural analysis, enzyme activity, as well as the accumulation of proteins related to oxidative stress were analyzed.

## Materials and Methods

### Plant material and cultivation conditions

The experiment was conducted in a greenhouse. Two progenies of *T*. *cacao*, were used. One progeny was a result from the cross between *CCN-10 x SCA-6*. CCN-10 is resistant to biotic and abiotic stresses [41,42], and SCA-6 is a clone often used in genetic improvement for being resistant to many types of stresses [43]. The other progeny was obtained from the self-pollination of *Catongo*, which is highly susceptible to various biotic stresses [44,45].

The progenies were obtained from controlled pollination of flowers from 5 to 10 year old cocoa trees belonging to the Germplasm Cacao Collection of the Centro de Pesquisas do Cacau (CEPEC, 39°13'59"W, 14°45'15"S, 55 m asl), of the Comissão Executiva do Plano da Lavoura Cacaueira (CEPLAC), Ilhéus, Bahia, Brazil. The permission to use accessions from the germplasm collection was granted by the cacao geneticist and researcher Wilson Reis Monteiro from CEPEC/CEPLAC. Field studies did not involve endangered or protected species, since *T*. *cacao* is a cultivated species.

After fruit maturation and collection (about six to seven months after anthesis), the seeds were removed. Then, the pulp/mucilage was eliminated by using sawdust. The integument surrounding the seeds was also removed. Afterwards, the seeds were soaked in solutions with increasing concentrations of Pb (0, 0.05, 0.1, 0.2, 0.4, and 0.8 g L^-1^), in the form of PbNO_3_, during 24 hours. Shortly after the period of soaking, the seeds, already in the process of germination, were transferred to black conical plastic tubes of 235 cm^3^ containing organic substrate (ground *Pinus* bark + coconut fiber in the ratio of 1:1), enriched with mineral macro and micronutrients, and irrigated daily with demineralized water. The emergence of the seedlings began approximately seven days after sowing. From the 30^th^ day after the emergence (DAE), the seedlings were fertilized weekly with 5 mL/tube of the solution containing 4 g of NH_4_H_2_PO_4_, 3 g of (NH_2_)_2_CO, and 3 g of KNO_3_ per liter of demineralized water until the collection of the seedlings, which occurred at 60 DAE, at which point all the cotyledons had already fallen.

### Ultrastructural analysis of cell organelles of roots and leaves by transmission electron microscope (TEM)

Samples of roots and leaves of seedlings of two progenies of *T*. *cacao*, subjected to different concentrations of Pb (control, 0.2, and 0.8 g Pb L^-1^) and three replicates for each treatment were collected and fixed in 2.5% glutaraldehyde, in a sodium cacodylate buffer at 0.1 M, pH 6.8, during 4 hours. The samples were then subjected to a series of washes in a sodium cacodylate buffer at 0.1 M, pH 7.2, and post-fixed in 1% osmium tetroxide, prepared in the same buffer, during 2 hours at 4°C. Subsequently, the samples were dehydrated in an increasing ethanol series (30, 50, 70, 80, and 90%), followed by two washes in 100% ethanol. Soon after, the samples were soaked in a mixture of 100% ethanol and LR White resin in the proportions of 3:1 (2 h), 1:1 (2 h), 1:3 (overnight), followed by two changes of pure LR White resin every 4 h, always under slow agitation. Afterwards, the samples were placed in gelatin capsules and covered with pure LR White resin. Resin polymerization was completed in 24 hours at 60°C. The ultrathin sections (60–70 nm) were made with a diamond knife, using a Leica ultramicrotome (model UC6, Nussloch, Germany). The cut sections were deposited on copper grids, contrasted with uranyl acetate in aqueous solution for 25 min, and then with lead citrate for 30 min [46]. Subsequently, they were observed in a transmission MORGANI electron microscope (FEI Company, model 268 D, Eindhoven, Netherlands).

### Thiobarbituric acid reactive substances

The extraction of thiobarbituric acid reactive substances (TBARS) was performed according to the Protocol described by Heath and Packer [47]. Samples of root and leaves of seedlings of the two progenies of *T*. *cacao* were used, subjected to different concentrations of Pb (control, 0.05, 0.1, 0.2, 0.4, and 0.8 g Pb L^-1^), and three replicates for each treatment were made. The accumulated concentration of TBARS was determined by means of reading of the absorbance of the reactions at 532 nm.

### Guaiacol Peroxidase (GPX, E.C. 1. 11. 1.7)

For analysis of the activity of the guaiacol peroxidase (GPx), roots and leaves of seedlings of *T*. *cacao* from both progenies previously subjected to the treatments with different concentrations of Pb (0, 0.05, 0.1, 0.2, 0.4, and 0.8 g L^-1^) with three replicates for each treatment were collected at 60 DAE, frozen in liquid nitrogen, and stored in an ultrafreezer at -80°C until the point of lyophilization. The enzymatic extract was obtained according to the protocol described by Rehem et al. [42]. For the enzymatic assay, 96-well Microplates containing 140 μL of reaction buffer POD 2 x [40 mmol L^-1^ of guaiacol, H_2_O_2_ at 0.06% and sodium phosphate (20 mmol L^-1^, pH 6.0)], 139 μL of phosphate buffer (50 mmol L^-1^, pH 6.0), and 1 μL of enzyme extract previously diluted, were used. The reading was conducted in a microplate spectrophotometer (VERSAmax). The guaiacol peroxidase activity was expressed with the increase in consumption of guaiacol in μmol h^-1^ g^-1^ of dry matter. The conversion of the obtained data to absorbance values at 470 nm min^-1^ g^-1^ of dry matter, for the consumption of guaiacol in mmol h^-1^ g^-1^ of dry matter, was performed according to the equation used by Rehem et al. [42].

### Gene expression

The RNA was extracted from leaves and roots in three different treatments (control, 0.2, and 0.8 g Pb L^-1^) with three replicates for each treatment. The RNA was extracted with the kit. The purity and the integrity of the RNA were tested by electrophoresis in 1% agarose gel. The RNA samples were used for cDNA synthesis using Revertaid H-Minus Reverse Transcriptase, according to the manufacturer’s instructions. The reactions were incubated at 65°C for 5 min, 37°C for 5 min, 42°C for 60 min, and 70°C for 10 min.

Real-time quantitative relative PCR (qPCR) was carried out in a thermal cycler “Real Time PCR” (Applied Biosystems, model 7500, Foster City, USA) using non-specific detection sequence (fluorophore), SYBR Green I. The abundance of transcripts was analyzed by means of specific primers as presented in Table 1 of the genes that encode for phytochelatin synthase, guaiacol peroxidase, and superoxide dismutase, designed from the analysis of gene sequences from the cocoa library (http://cocoagendb.cirad.fr).

**Table 1:** Pairs gene-specific primers that were used in qRT-PCR analysis.

PCs	XM_007050160	Heavy metal-detoxifying	TcPCs F 5’- TTCAGGCACGGTAATTAGTAATGG -3’
			TcPCs R 5’- GGATGCATGCCACAACAATTAT -3’
Cu-ZnSOD_Cyt_	CL94Contig1	Biosynthesis of cytosolic	Cytopl CuZnSOD F 5’TGATGGCTGTGTGAGTTTCTCT 3’
		Cu-ZnSOD^b^	Cytopl CuZnSOD R 5’AACAGCTCTTCCAATAATTGA3
PER-1	CK144296.1	Biosynthesis of peroxidase	
			TcPER F 5’ CAGGTGTCGTGGGATCAAGA 3’
		class III^a^	TcPER R 5’ TGGAAAAACTACGCCAAATATGC
*β* -Tubulina	GU570572.1	Endogen^c^	β -Tub F 5’-TGCAACCATGAGTGGTGTTCA- 3’
			β -Tub R 5’-CAGACGAGGGAAGGGAATGA-

^a^ http://cocoagendb.cirad.fr/

^b^ http://esttik.cirad.fr/index.html

^c^ http://www.ncbi.nlm.nih.gov/.

The reaction mixture consisted of: cDNA (500 ng) template, 0.5 uM of each initiator, and 10 μL fluorophore SYBR Green I (Fermentas, Pittsburgh, USA) in a final reaction volume of 20 μL. The temperature of the PCR products was raised from 55 to 99°C at a rate of 1°C/5s, and the resulting data analyzed using the LightCycler software. Only a single band with a characteristic melting point was observed for each sample, indicating that the qPCR produced a specific product produced by the initiators used. Threshold Cycle (TC) values were determined using the LightCycler software. The relative expression of genes was calculated as a percentage of the control progenies, using the method 2^-ΔΔCt^ set out by Livak and Schmittgen [48] and β-Tubulin as endogenous controls in order to detect alterations in the abundance of transcripts (Table 1). All the reactions were prepared in triplicates and performed twice. Three biological replicates were used for each assessment.

### Mineral nutrients

Roots, stems, and leaves of the two progenies were collected and subjected to different concentrations of Pb (control, 0.05, 0.1, 0.2, 0.4, and 0.8 g Pb L^-1^) and five replicates for each treatment, were performed. The collected material was washed 1x in tap water, 1x in HCl at 3%, and 2x with deionized water. The different plant organs were then placed in an oven at 75°C until a constant weight was obtained in order to calculate dry biomass. Afterwards, the dried plant organs were ground with a Wille mill (Thomas Scientific, Swedesboro, USA) using 20-mesh screens, and then chemically analyzed according to the methodological procedures described by Anunciação et al. [49]. The concentrations of mineral nutrients were evaluated in relation to the dry matter of the roots, stems, and leaves by using the technique of inductively coupled plasma optical emission spectrometry (ICP-OES), Model Varian 710-ES.

### Proteomic analysis

#### Protein extraction

Samples of roots and leaves of seedlings of the two progenies of *T*. *cacao*, from seeds germinated in high concentration of Pb (0.8 g L^-1^) and in the absence of Pb, collected at 60 DAE, were obtained by extraction with phenol, followed by precipitation with ammonium acetate at 0.1 M in methanol, as described by Pirovani et al. [50], and adapted for roots in accordance to Bertolde et al. [51]. Three replicates for each treatment were conducted.

#### Two-Dimensional SDS-PAGE

For the two-dimensional gel, the first dimension was conducted in an Ettan IPGphor system (GE Healthcare). The sample of protein (350 ng) was applied to 250 μL of rehydration solution, along with strip type Immobiline DryStrip Reswelling (pH 3–10, GE Healthcare) of 13 cm, during 12 h and, subsequently, focalization was conducted in the same device.Afterwards, the strips were stored at -80°C until the analysis of the second dimension. Before making the SDS-PAGE gel, the strips were incubated for 15 min. in a buffer solution of equilibrium [urea at 6 M, Tris-HCl (7.5 mM and pH 8.8), 29.3% glycerol, 2% SDS, and bromophenol blue at 0.002%] and with DTT at 1% (p/v), for another period of 15 min., in a buffer of equilibrium with iodoacetamide at 2.5% (w/v). The second dimension (SDS-PAGE) was executed in a SE600 Ruby system (GE Healthcare): 15 mA, during 45 min, 40 mA, during 30 min., and 50 mA per gel, for 3 hours, for each strip, at a constant temperature of 11°C. The molecular weight marker used was the GE Healthcare. After the electrophoresis, the proteins were stained with colloidal coomassie at 0.08% w/v of G-250. The gels were scanned via *ImageScanner II (Amersham)* and analyzed using the ImageMaster Platinum 2D 6.0 software (GE Healthcare).

#### Mass spectrometry

The selected protein spots were removed from the two-dimensional gel, balanced with acetonitrile at 50%, containing 25 mM ammonium bicarbonate, to remove the blue stain from the coomassie and then rinsed with distilled water. The digestion of proteins was performed according to Silva et al. [52]. The eluted peptides were directly introduced to a mass spectrometer Micromass Q-TOF Micro System (Waters, Manchester, United Kingdom) through its electrospray membrane probe. The most abundant ions observed in the spectrum of MS were automatically selected for collision-induced dissociation, using the Masslynx software, generating MS/MS spectra. Gaseous argon was used for the collision-induced dissociation peptide. The resulting spectra were processed by the MaxEnt3 algorithm of the Masslynx ProteinLynx software to generate a list of masses corresponding to the peaks of the spectra obtained in the analysis. The list of the peaks generated by Proteinlynx 2.4 was searched for in *T*. *cacao* genome databases and NCBI. In this research, the 2.1.0 version of the MASCOT (Matrix Science), was used. The identification was performed by Mass Fingerprint Peptide and sequenced by means of MS/MS.

### Statistical analysis

The experimental design used was the completely randomized design, with five replicates of 50 seeds, in a 2 x 6 factorial scheme, composed of two progenies of *T*. *cacao* (CCN-10 x SCA-6 and *Catongo*) and six concentrations of Pb (0, 0.05, 0.1, 0.2, 0.4, and 0.8 g L^-1^). Analysis of variance (ANOVA), comparison of means (intraprogenic x doses) by the Tukey’s test (p<0.05), and comparison of means using the t-test (p<0.05), were conducted. Additionally, regression analysis for the mineral micronutrients, was also performed.

## Results

### Ultrastructural analyses of the foliar and root mesophyll

**Fig 1:**
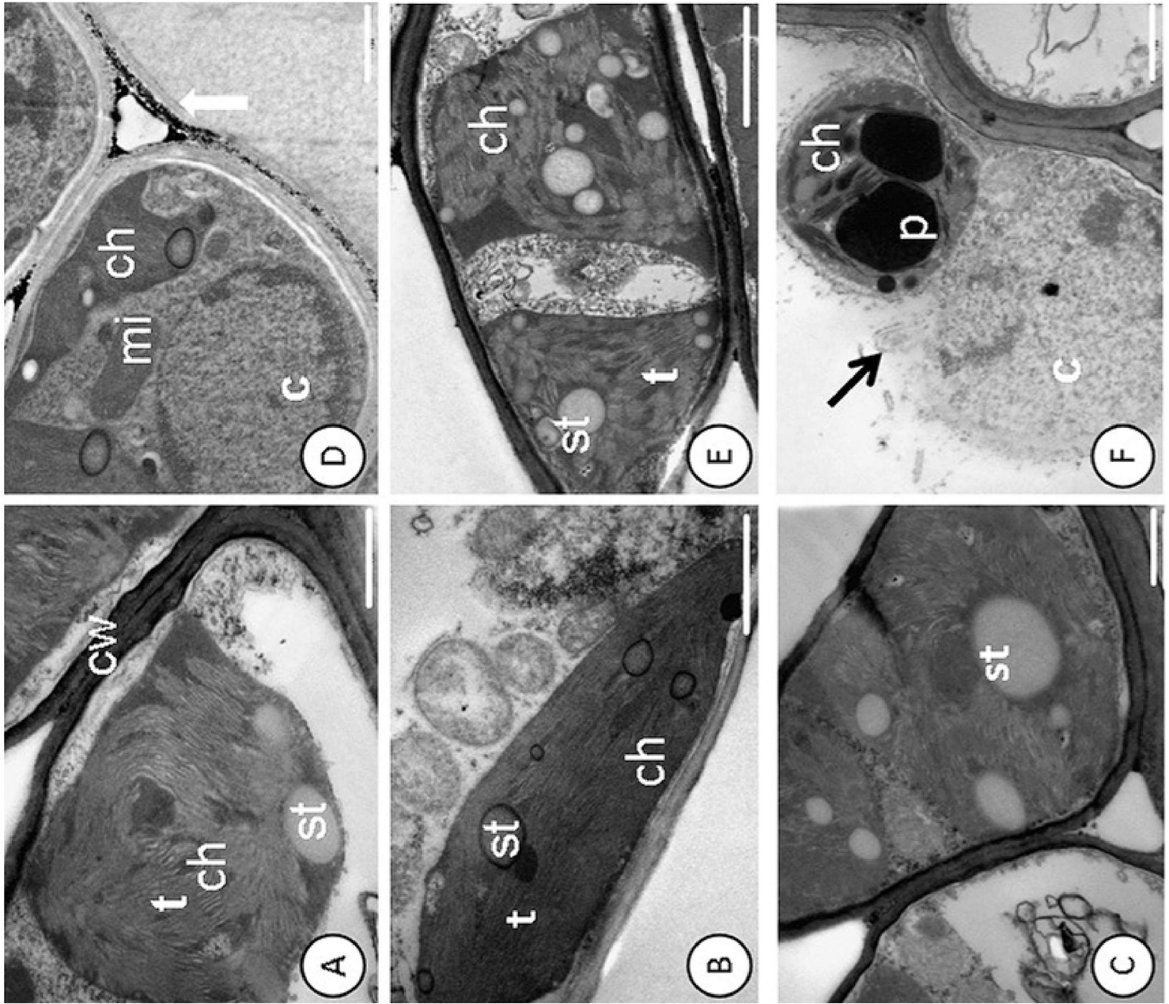
Disruption of the nuclear membrane and deposition of electro-dense material in the cell wall detected by ultrastructural micrographs of leaf mesophyll cells. Catongo control **(A), (B)** and **(C)** and submitted to dose of 0.8 g Pb L^-1^ **(D)**, **(E)** and **(F)**. st—starch; ch—chloroplast; mi—mitochondria; p—plastoglobule; t–thylakoid; black arrow—breakup of the nucleus; white arrow—deposition of electron-dense material. Bars: 1.0 mm.

**Fig 2:**
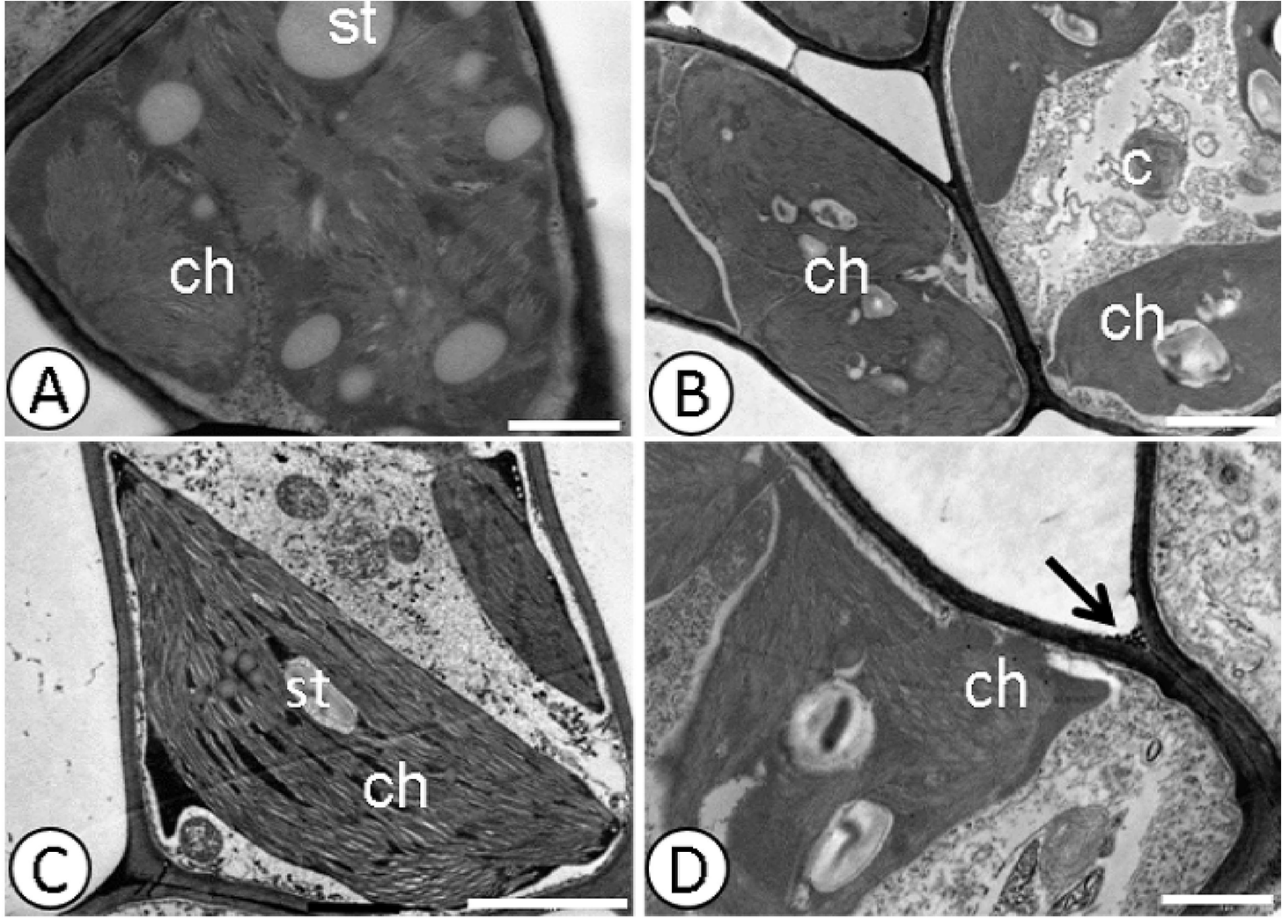
Deposition of electro-dense material in the cell wall detected by ultrastructural micrographs of leaf mesophyll cells. CCN-10 x SCA-6 Control **(A)** and **(C)**, and submitted to dose of 0.8 g Pb L^-1^ **(B)** and **(D)**. st—starch; ch—chloroplast; p—plastoglobule; t—thylakoid; Arrow-deposition of electro-dense material. Bars: 1.0 mm.

**Fig 3:**
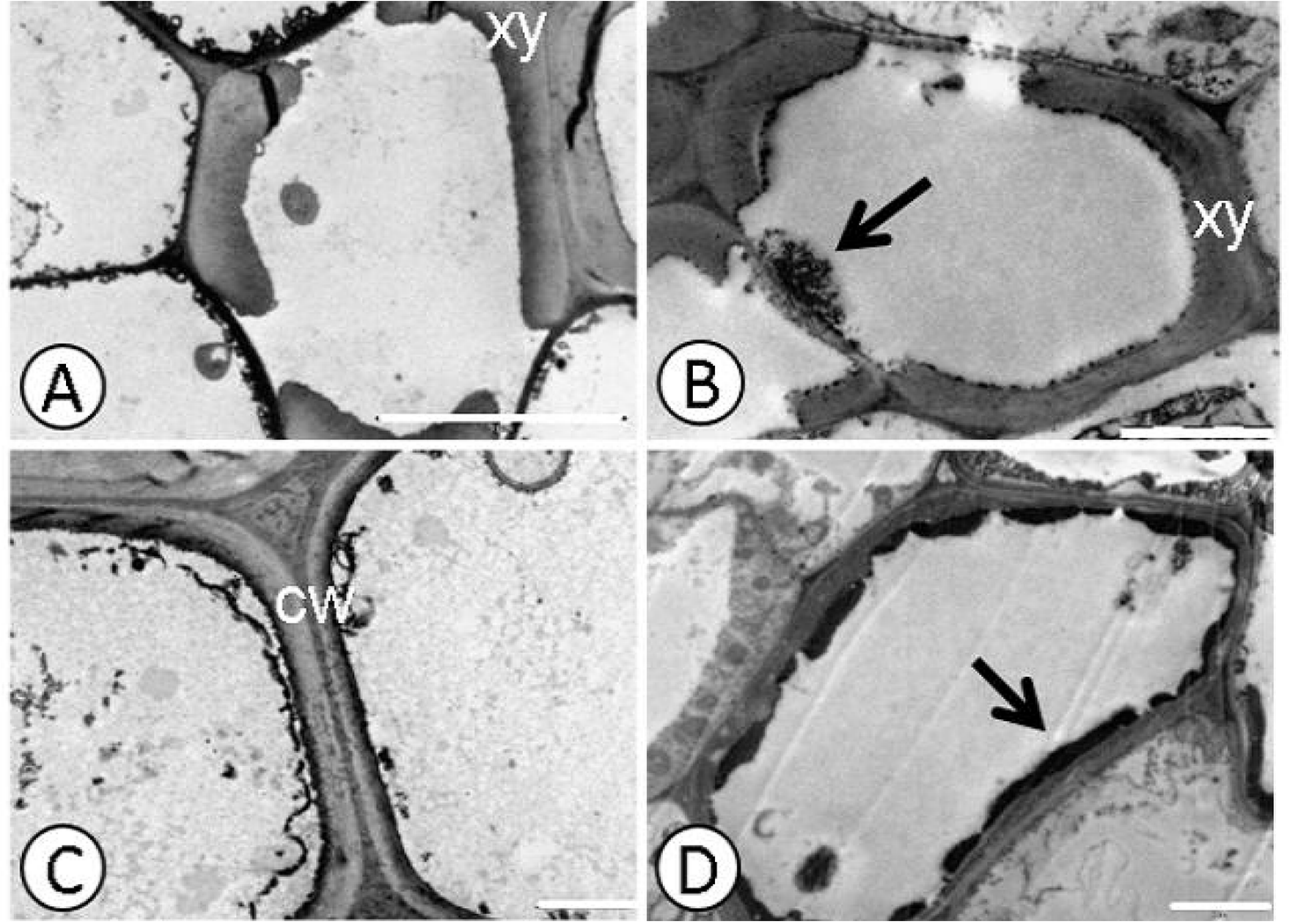
Deposition of electro-dense material in xylem and endoderm detected by ultrastructural micrographs of root cells. Catongo control **(A)** and submitted to dose of 0.8 g Pb L^-1^ **(B).** CCN-10 x SCA-6 control **(C)** and subjected to a dose of 0.8 g Pb L^-1^ **(D)**. cw—cell wall; xy–xylem; arrow-deposition of electro-dense material. Bar: 1 μm.

**Fig 4:**
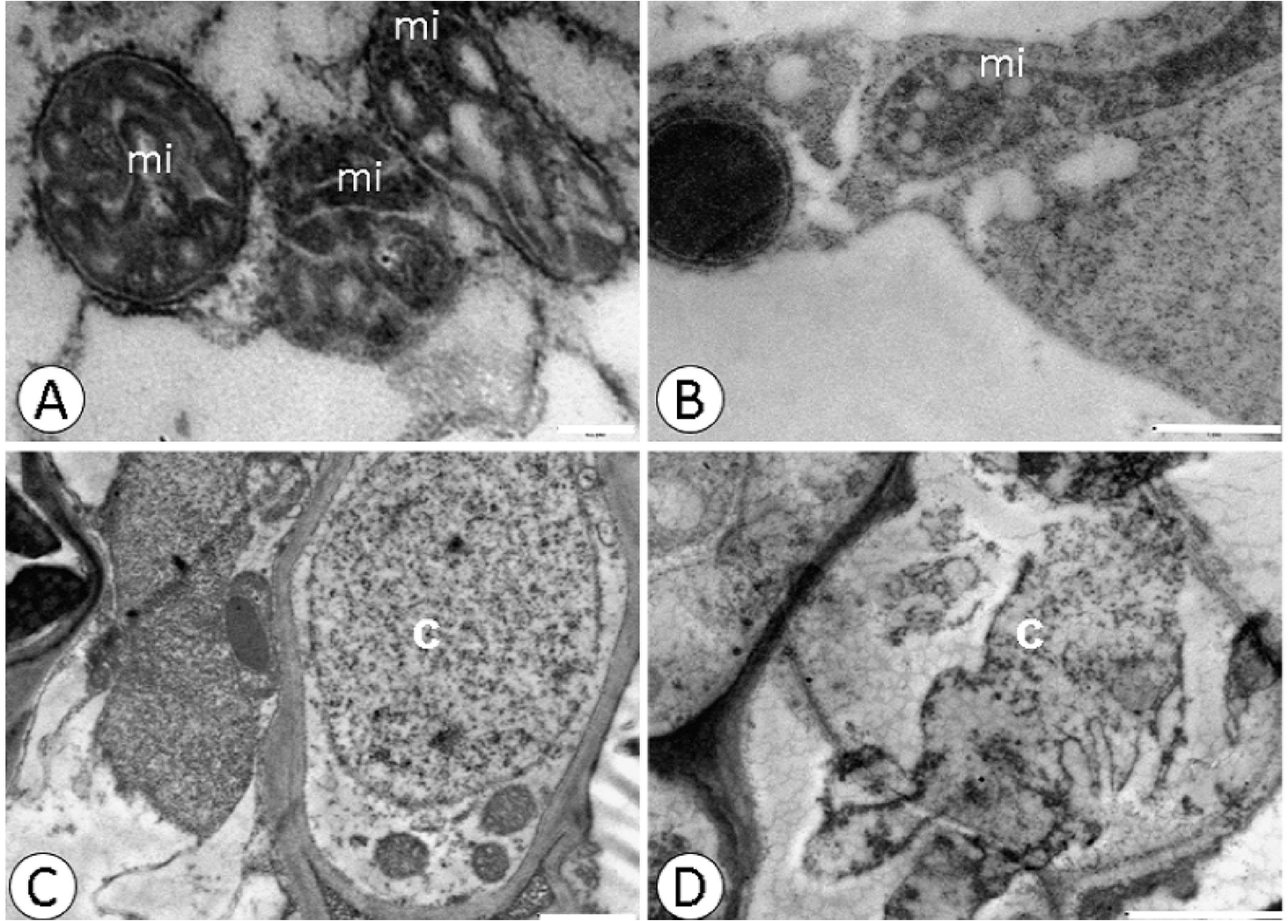
Changes in the nucleus and mitochondria detected by ultrastructural micrographs of root tissue cells. CCN-10 x SCA-10 control **(A)** and **(C),** and submitted to dose of 0.8 g Pb L^-1^ **(B)** and **(D)**. c-core; mi-mitochondria; p- plastoglobule. Bars: 0.2 μm (A); 1μm (B and C); 0 2 μm (D).

Pb caused changes in the cell ultrastructure of the foliar mesophyll in the susceptible progeny (*Catongo*), when subjected to the dose of 0.8 g Pb L^-1^ via seminal. Disorganization in tilacoidal membranes, poorly developed chloroplasts (Fig 1E), and rupture of the nuclear membrane (Fig 1F), were verified in this progeny. In both progenies, electrodense deposits were observed between the cell walls of the foliar mesophyll (Figs 1D and 2D). *Catongo* and CCN-10 x SCA-6, in the absence of Pb, presented cells of the foliar and radicular mesophyll with normal aspect (Figs 1A–1C; 2A and 2C; 3A and 3C, and 4A and 4C).

Electrodense deposits were verified within the xylem cells of the susceptible progeny and within the endoderm of the resistant progeny (Fig 3B and 3D), when subjected to a dose of 0.8 g Pb L^-1^. Pb caused alterations in the mitochondria and rupture of the nuclear membrane in root cells (Fig 4B and 4D) of the resistant progeny.

### Thiobarbituric acid reactive substances

**Fig 5:**
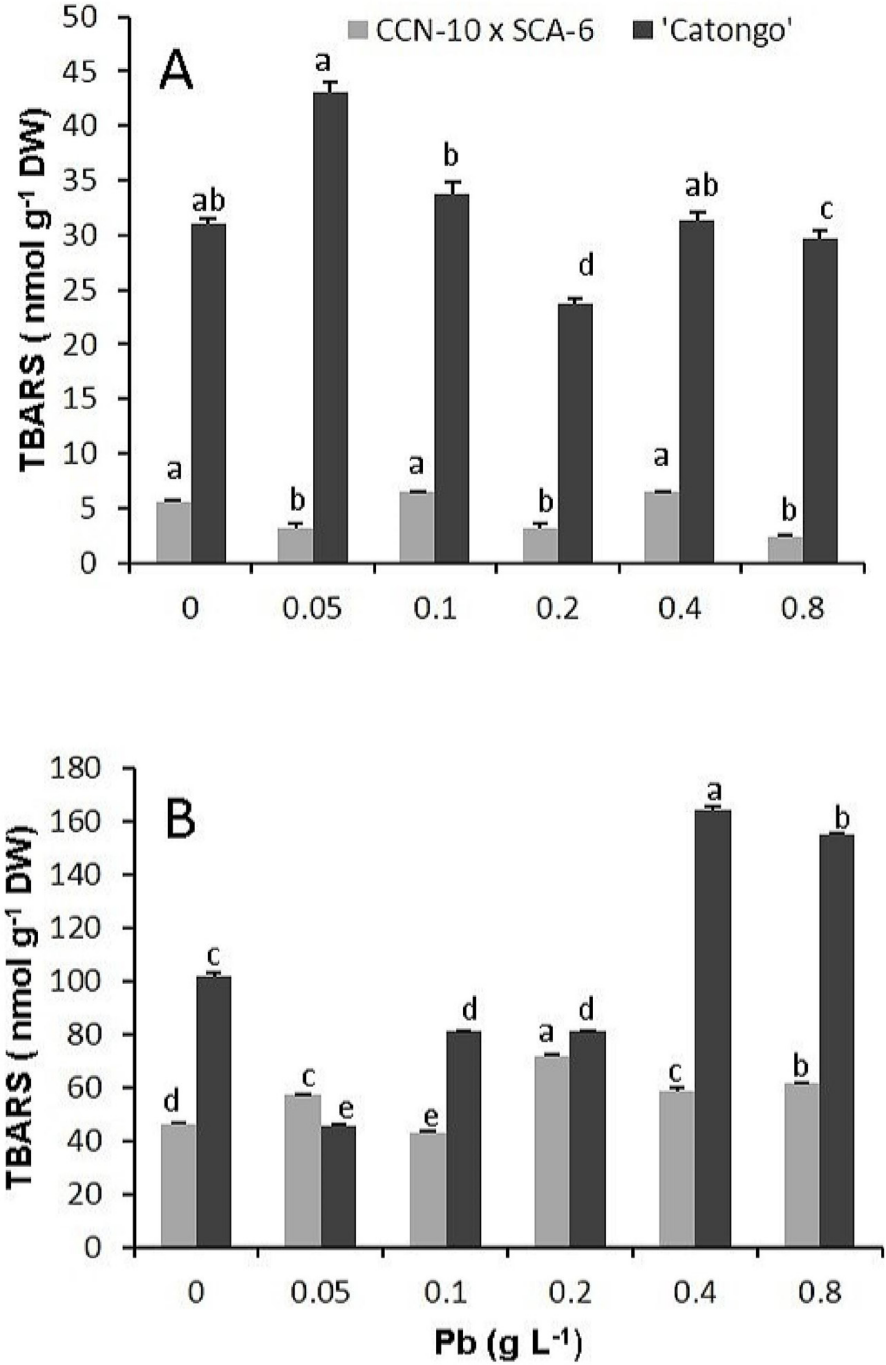
Concentration of thiobarbituric acid reactive substances (TBARS). Leaves **(A)** and roots **(B)** of two progenies of *T*. *cacao* exposed to increasing doses of Pb. Mean values intraprogenies followed by the same lowercase letters do not differ by Tukey test (p<0.05). Mean values of four replicates (± SE).

The resistant progeny showed no significant difference (p< 0.05) for leaf accumulation of TBARS compared to the controls (0 g Pb L^-1^). However, showed a significant increase (p< 0.05) of TBARS in root of 0.6, 0.3 and 0.3 times for the doses corresponding to 0.2, 0.4, and 0.8 g Pb L^-1^, respectively, compared to controls. Already susceptible progeny showed an increase of 0.4 times of TBARS in the leaves, at a dose of 0.05 g Pb L^-1^ in comparison to the control (Fig 5 S1 Table).

### Guaiacol peroxidases

**Fig 6:**
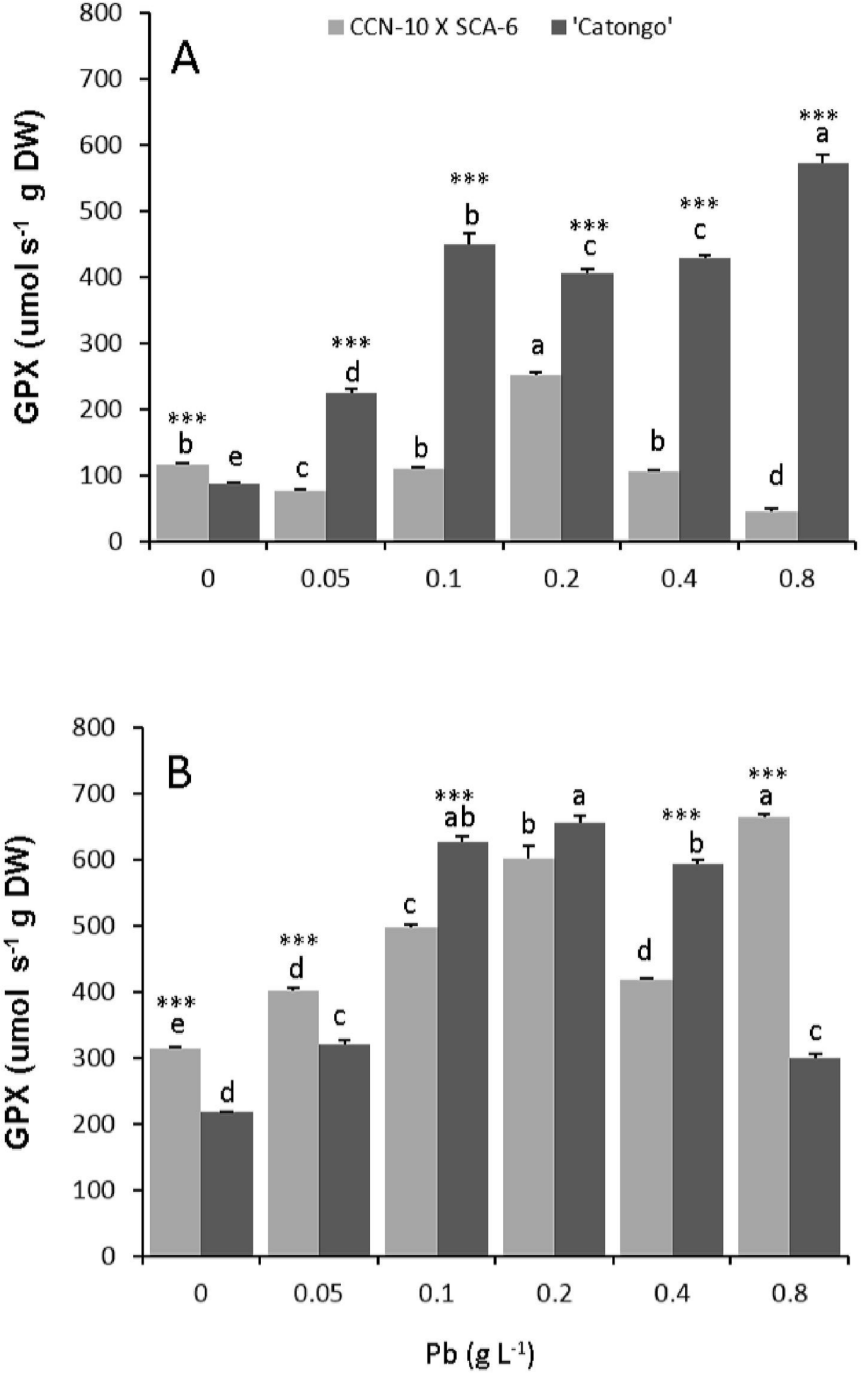
Guaiacol peroxidase activity (POD). Leaves **(A)** and roots **(B)** of two progenies of *T*. *cacao* exposed to increasing doses of Pb. The statistical significance interprogenies was obtained by t-test. (*) p<0.05; (**) p<0.01; (***) p<0.001; (ns) not significant. Mean values intraprogenies followed by the same lowercase letters do not differ by Tukey test (p<0.05). Mean values of four replicates (± SE).

The susceptible progeny presented a significant increment (p<0.05) in the leaves of 1.6, 4.1, 3.6, 3.9, and 5.5 times for the doses corresponding to 0.05, 0.1, 0.2, 0.4, and 0.8 g Pb L^-1^, respectively, in comparison to the control (Fig 6A, S2 Table). Already resistant progeny presented a significant increase of 1.2 times for the GPX activity only for the dose of 0.2 g Pb L^-1^. In the roots of the two progenies, there was an increase of GPX activity parallel to the increase in doses of Pb (Fig 6B). The susceptible progeny presented an increase of 0.5, 1.9, 2.0, 1.7, and 0.4 times in the GPX activity for the doses of 0.05, 0.1, 0.2, 0.4, and 0.8 g Pb L^-1^, respectively, in comparison to the control. Regarding the resistant progeny, there was an increase of 0.3; 0.6; 0.9, 0.3 and 1.1 times for the doses of 0.05, 0.1, 0.2, 0.4, and 0.8 g Pb L^-1^, respectively, in comparison to the control.

### Gene expression

**Fig 7:**
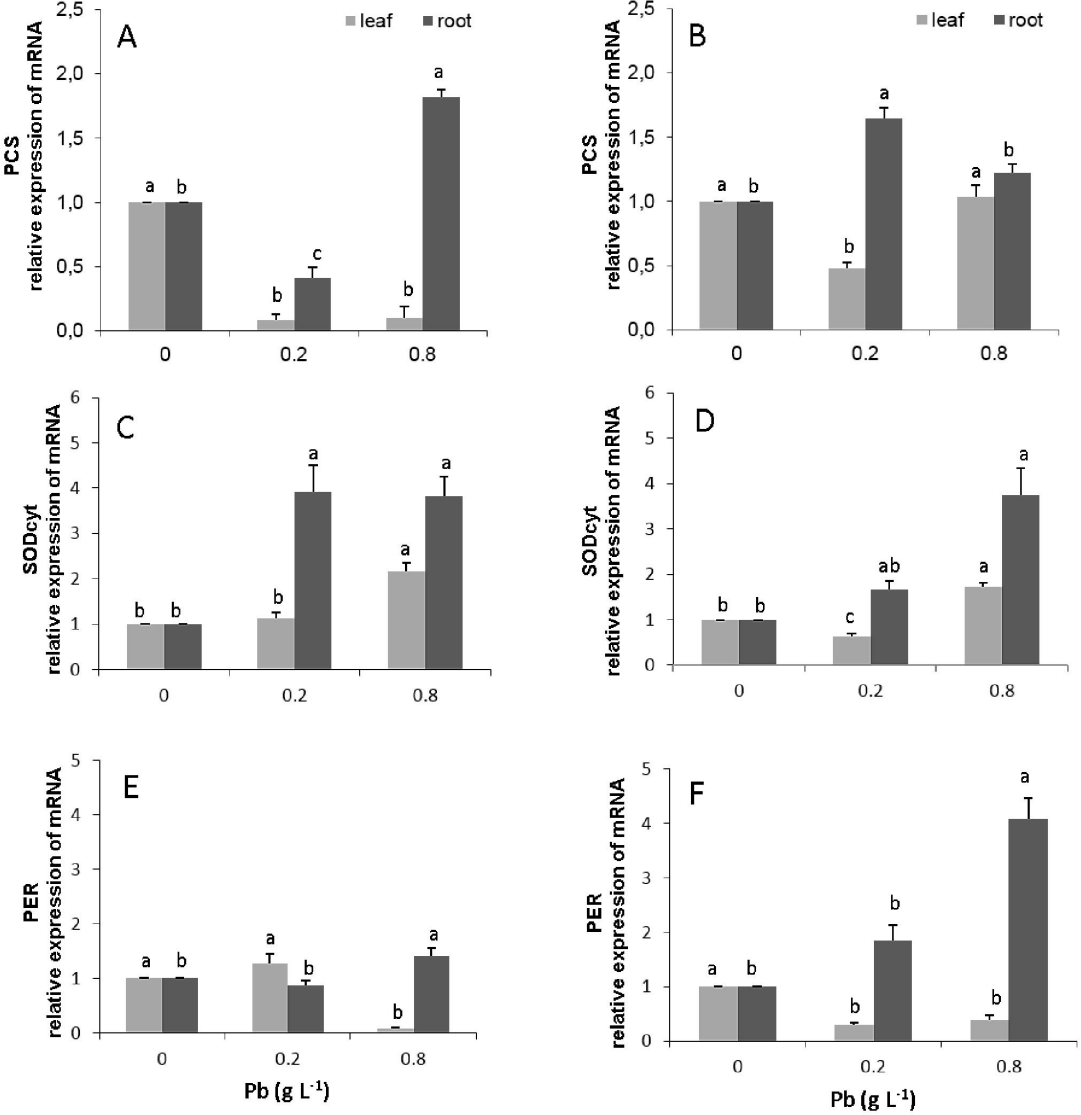
Amount of gene transcripts of the biosynthetic pathway of phytochelatins synthase (PCs); cytoplasmic superoxide dismutase (SODCyt) and peroxidase (PER-1) in leaves and roots of *T*. *cacao* progenies. Catongo **(A)**, **(C)** and **(E),** and CCN-10 x SCA-6 **(B)**, **(D)** and **(F)** exposed to increasing of Pb doses. The mRNA levels were quantified by quantitative real-time PCR. The mRNA levels were normalized in respect to tubulin, and are expressed relatively to those of control plants that were given a value of 1. Mean values of Intraprogenies followed by the same lowercase letters do not differ by Tukey test (p <0.05). Mean values of six replicates (± SE).

The transcripts from the gene phytochelatin synthase (PCS) were not detected in leaves in both progeny. In the roots, the susceptible progeny presented an increase in expression of this gene of about 0.8 times for the dose of 0.8 g Pb L^-1^ (Fig 7A, S5 Table). In contrast, in the resistant progeny, there was an increase in the expression of 0.7 and 0.2 times for the doses of 0.2 and 0.8 g Pb L^-1^, respectively (Fig 7B).

The resistant and susceptible progenies presented an increase in the expression of the SODcyt gene in leaves only for the higher dose of Pb (0.8 g L^-1^), corresponding to 0.7 and 1.2 times, respectively (Fig 7C and 7D, S3 Table). However, in the roots of the susceptible progeny, there was an increase of 3.2 and 2.8 times in the expression of the SODcyt gene for the doses of 0.2 and 0.8 g of Pb L^-1^, respectively (Fig 7C). In contrast, for the resistant progeny, the increases for these same doses were of 0.7 and 2.2 times, respectively (Fig 7D).

The PER-1 gene presented a significant increase of 0.4 times in its expression in the susceptible progeny, only for the roots and for the highest dose. In the resistant progeny, there was a significant increase (p<0.05) in the expression of PER-1 only in the roots, with an increase of 1.8 and 4.0 times for the doses of 0.2 and 0.8 g Pb L^-1^, respectively (Fig 7F, S4 Table).

### Contents of Pb and macro and mineral micronutrients

**Fig 8:**
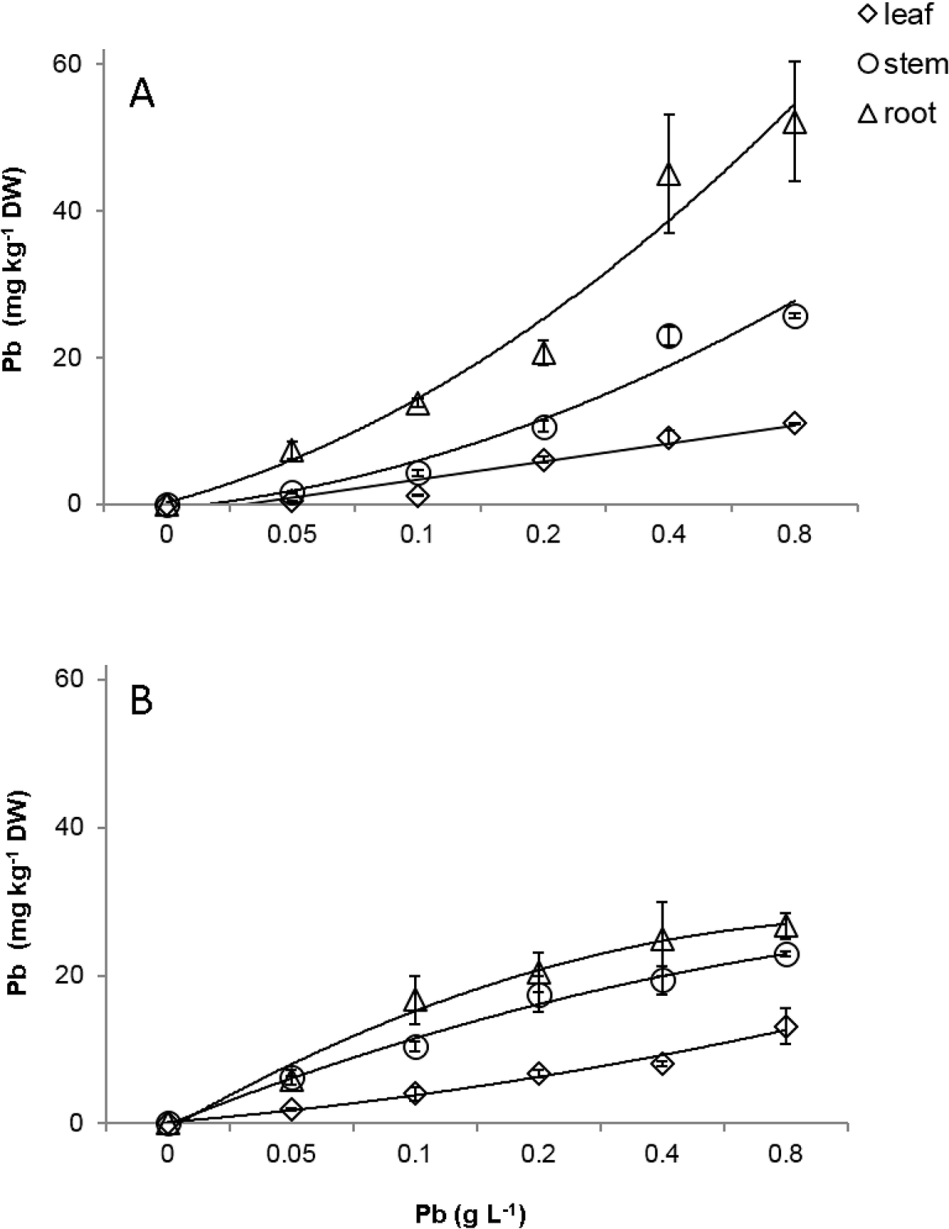
Accumulation of Pb in roots (triangle), stem (circle) and leaves (rhombus) of two progenies of *T*. *cacao* submitted to increasing of Pb doses. CCN-10 x SCA-6 **(A)** and Catongo **(B)**. Mean values of nine replicates (± SE). The equations of the regression curves were: CCN-10 x SCA-6: ŷ = - 3.99 + 2.46*x (R² = 0.92) for leaf, ŷ = - 9.02 + 5.68*x, (R² = 0.92) for stem, ŷ = -17.58 + 11.74*x (R² = 0.87) for root. Catongo: y = - 3,047 + 2.49*x (R² = 0.96) for leaf, ŷ = -10.43 + 10.43*x– 0.88 x^2^ (R² = 0.94) for stem, ŷ = - 0.92 + 5.68 ln*(x) (R² = 0.93) for root.

Pb was detected in the roots, stems and leaves of the susceptible and resistant progenies. The Pb concentrations found in the different organs were proportional to the increase of the doses of Pb supplied via seminal for both progenies (Fig 8A and 8B). The highest accumulation of Pb was evidenced in the roots of the resistant progeny at 60 days after emergence (DAE) (Fig 8B, S6 Table). The roots, stems and leaves of the susceptible and resistant progenies accumulated 24.7, 19.9, and 13.1 mg Pb kg^-1^ DW and 52.1, 25.7, and 11.0 mg Pb kg^-1^ DW respectively, in the dose corresponding to 0.8 g Pb L^-1^.

**Fig 9:**
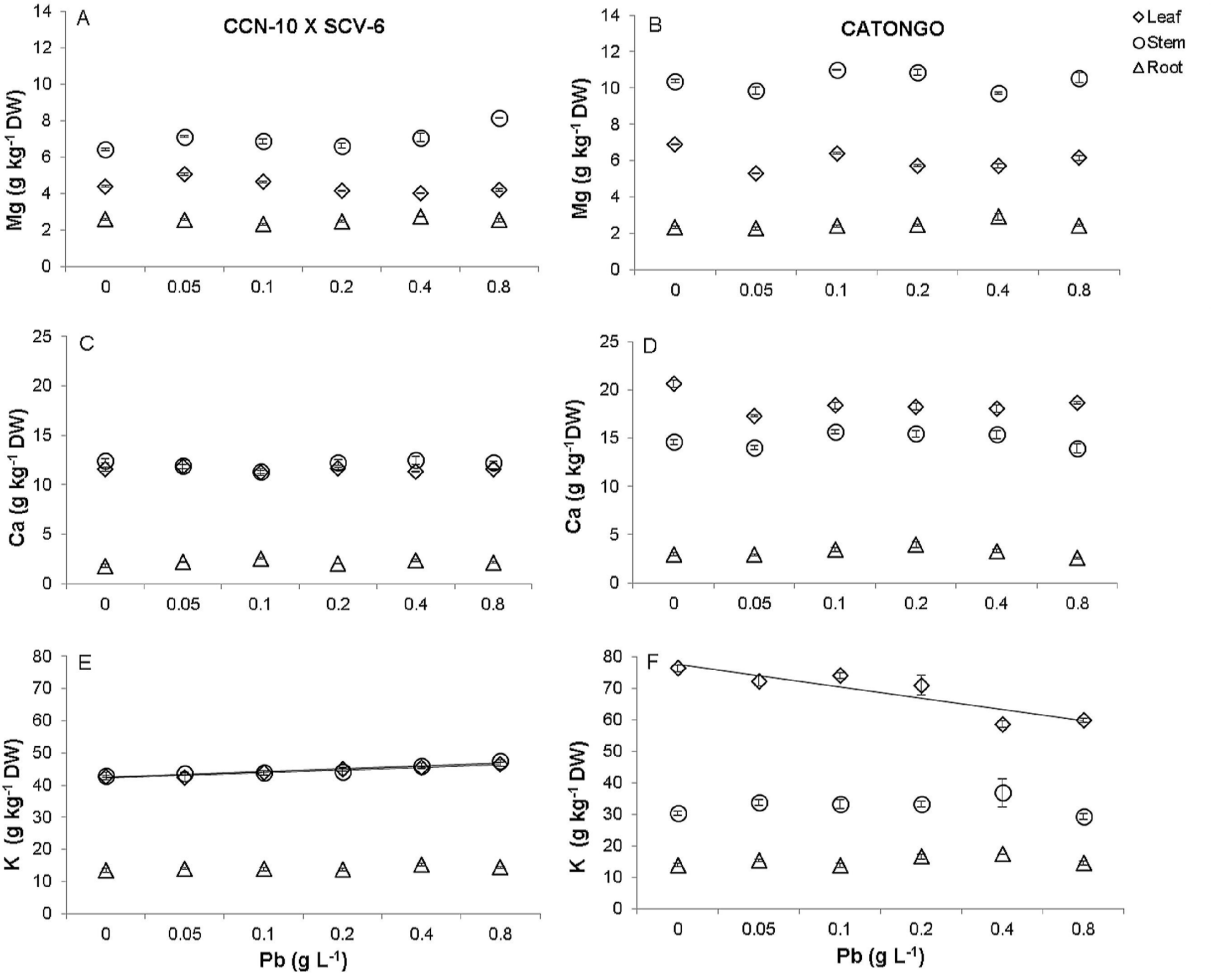
Accumulation of mineral macronutrients (Mg, Ca, K) in roots (triangle), stem (circle) and leaves (rhombus) of two progenies of *T*. *cacao* exposed to increasing of Pb doses. CCN-10 x SCA-6 **(A,C, e E)** and Catongo **(B,D,e F).** Mean values of nine replicates (± SE). The absence of error bars indicates that the size of the error does not exceed the size of the symbol. The equations of regression curves were: **Mg—**CCN-10 x SCA-6: ŷ = 54.41 for leaf, ŷ = 7.05 for stem, ŷ = 2.54 for root. Catongo: ŷ = 6.03 for leaf, ŷ = 10.39 for stem, ŷ = 2.47 for root. **Ca—**CCN-10 x SCA-6: ŷ = 11.56 for leaf, ŷ = 12.10 for stem, ŷ = 2.19 for root. Catongo: ŷ = 18.54 for leaf, ŷ = 14.84 for stem, ŷ = 3.20 for root. **K—**CCN-10 x SCA-6: ŷ = 42.24 +0.8097*x (R^2^ = 0.93) for leaf, ŷ = 42.46 +0.8796*x (R^2^ = 0.88) for stem, ŷ = 14.09 for root. Catongo: ŷ = 43.15–1.9433*x (R^2^ = 0.74) for leaf, ŷ = 32.78 for stem, ŷ = 15.21 for root

**Fig 10:**
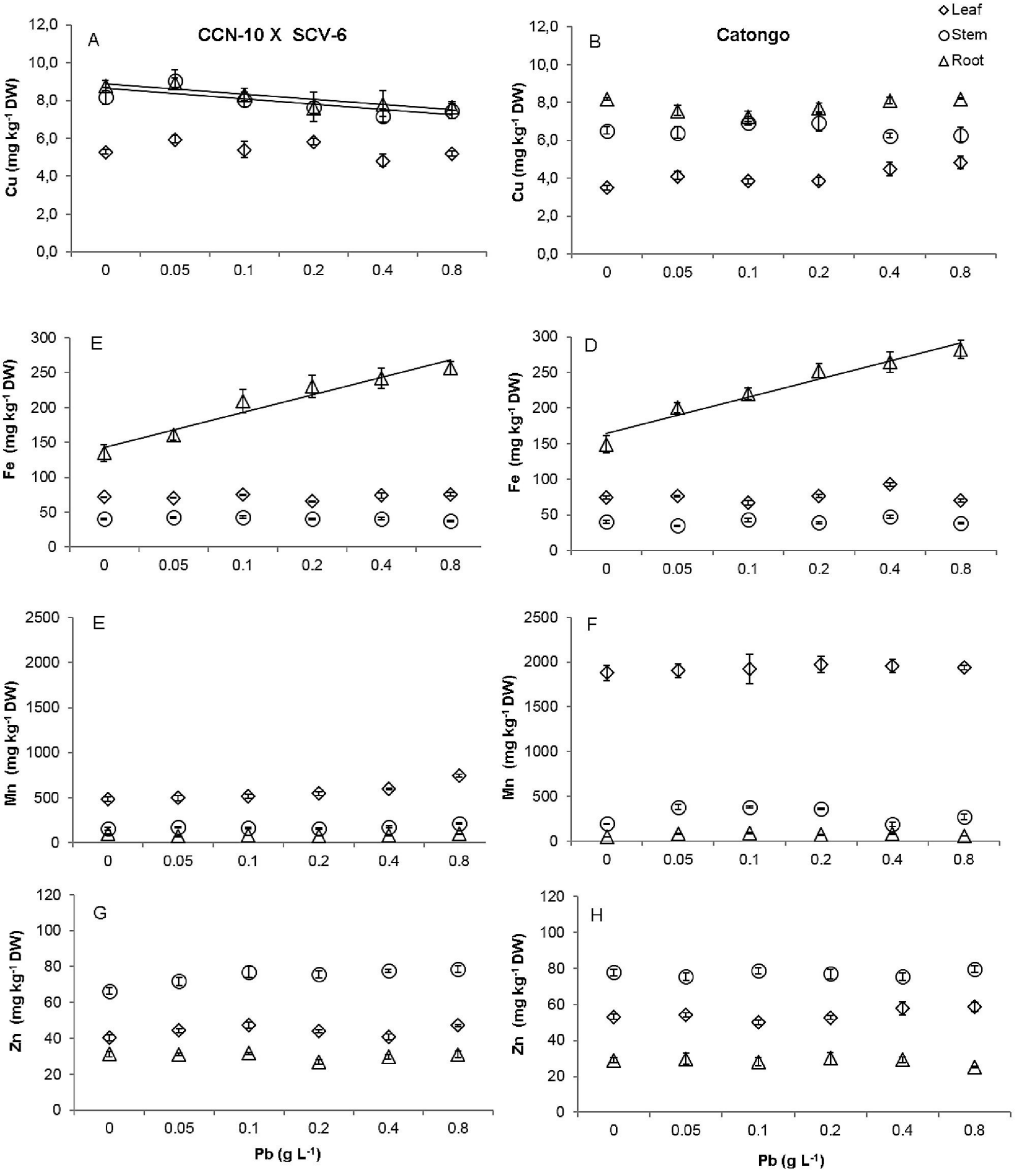
Accumulation of mineral micronutrients (Cu, Fe, Mn and Zn) in roots (triangle), stem (circle) and leaves (rhombus) of two progenies of *T*. *cacao* exposed to increasing Pb doses. CCN-10 x SCA-6 **(A,C,E e G)** and Catongo **(B,D,F e H).** Mean values of nine replicates (± SE). The absence of error bars indicates that the size of the error does not exceed the size of the symbol. The equations of regression curves were: **Cu—**CCN-10 x SCA-6: ŷ = 5.43 for leaf, ŷ = 8.65–0.278x (R^2^ = 0.51) for stem, ŷ = 8.88–0.2753*x (R2 = 0.74) for root. Catongo: ŷ = 4.11 for leaf, ŷ = 6.57 for stem, ŷ = 3.03 for root. **Fe—**CCN-10 x SCA-6: ŷ = 72.07 for leaf, ŷ = 40.86 for stem, ŷ = 118.10 + 24.97*x (R^2^ = 0.94) for root. Catongo: ŷ = 76.21 for leaf, ŷ = 40.36 for stem, ŷ = 138.90 + 25.49*x (R^2^ = 0.93) for root. **Mn—**CCN-10 x SCA-6: ŷ = 563.1 for leaf, ŷ = 173.5 for stem, ŷ = 84.1 for root. Catongo: ŷ = 1928.84 for leaf, ŷ = 295.4 for stem, ŷ = 72.52 for root. **Zn**—CCN-10 x SCA-6: ŷ = 44.5 for leaf, ŷ = 74.54 for stem, ŷ = 30.35 for root. Catongo: ŷ = 54.51 for leaf, ŷ = 77.43 for stem, ŷ = 28.51 for root.

There was a linear decrease in the concentration of Cu in the root and stem of the CCN-10 x SCA-6 progeny (Fig 10A). In contrast, the concentration of Fe increased 0.2 times in the roots of the CCN-10 x SCV-6 and *Catongo* progenies (Fig 10C and 10D). Furthermore, the concentration of K increased 0.1 times in the stem and leaf of the CCN-10 x SCA-6 progeny (Fig 9E, S7 Table), while for the *Catongo* progeny there was a linear decrease of 0.2 times for the concentration of K in the leaves (Fig 9F, S7 Table). However, there were no changes in Zn, Mn, Mg and Ca concentrations in the different organs of the T. cacao progenies (Figs 10E–10L and 9A–9D).

### Proteomics

**Fig 11:**
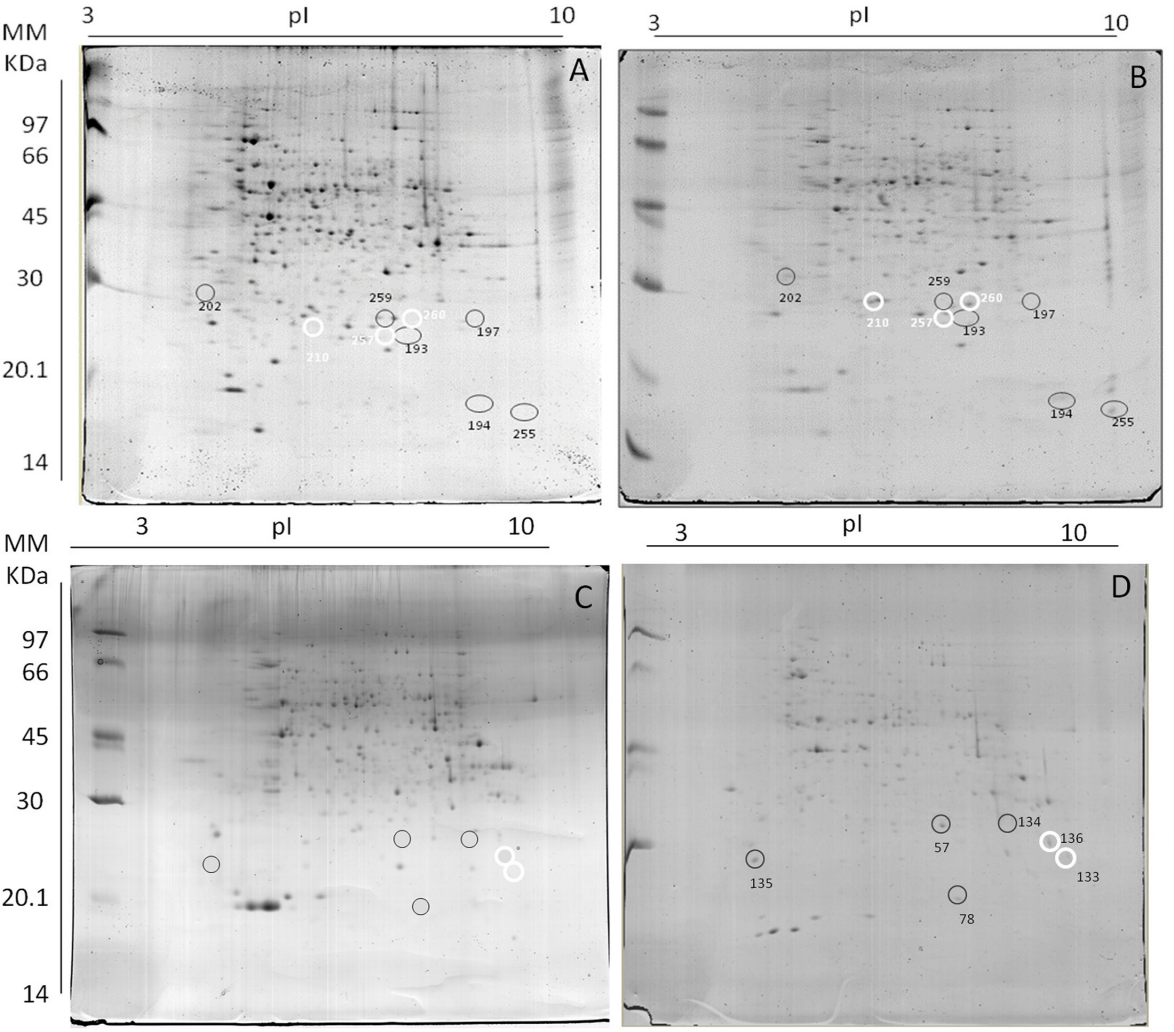
Two-dimensional gel analysis of proteins extracted from roots of *T*. *cacao* progenies. Catongo control **(A)** and submitted to 0.8 g Pb L^-1^ **(B)**. CCN-10 x SCA-6 control **(C)** and submitted to 0.8 g Pb L^-1^ **(D)**. Each gel was loaded with 350 ug of total protein stained with colloidal 0.08% Coomassie G-250. Black circles indicate single points detected among progenies in response to stress by Pb.

Analyses of the proteomic profile of the *T*. *cacao* progenies (susceptible and resistant) were performed by means of two-dimensional gel electrophoresis and mass spectrometry analyses. In both progenies, the proteins exclusively expressed, related to the responses to stress by Pb, were evaluated. In the susceptible progeny, 24 proteins were detected exclusively, when compared to the control (Fig 11A and 11B; Table 2). Among these, nine proteins were related to stress due to Pb. In the resistant progeny, 11 proteins were detected exclusively, with three related to heavy metal stress. In the susceptible progeny, it was possible to detect: peroxidase (PER), Spot 239; ascorbate peroxidase (APX), spot 210; glutathione-S-transferase (GST), spots 257 and 260; Osmotina (OSM), spot 197; aspartic protein, spot 202; Aldehyde dehydrogenase (ALDH), spot 229; and cytosolic NADP±-dependent isocitrate dehydrogenase (IDPc), spots 297 and 266. In the resistant progeny, it was possible to detect PER, spot 224; thaumatin, spot 142; and aspartic protein, spot 56 (Fig 11C and 11D; Table 3).

**Table 2:** Protein expression in roots of Catongo progeny identified by mass spectrometry.

Spot N°	Protein	MW(KDa)/pI	Biological process
70	Pyridoxal phosphate (PLP)dependent transferases superfamily protein	47.56/8.42	Metabolic process
	gi|508698790		
193	NAD- Glutamate dehydrogenase	15.45/9.43	Metabolic process
	Tc06 g003600		
194	Isomerase cis trans peptidil-prolil	18.19/8.12	Catalytic activity
	Tc02 g005490		
197	Osmotin	27.03/7.50	Plant defense
	Tc09 g031980		
202	Aspartic proteinase A1 isoform 1	59.75/4.86	Proteolysis
	gi|508701345		
210	L-Ascorbate Peroxidase cytosolic	32.65/7.31	Lipid metabolic process
	Tc09 g033010		
222	Protein	29.55/4.56	Metabolic process
	Tc04 g015130		
225	RNA binding protein rich in glycine	17.28 /8.75	Role in RNA transcription or processing during stress
	Tc02 g006970		
229	Aldehyde dehydrogenase family 2 member B4	62.27/7.63	Cellular metabolic process
	gi|508773422		
231	atp1 gene product	55.36/6.23	ATP synthesis
	gi|377806977		
237	Enolase	46.9/5.77	Glycolysis
	gi|508716212		
238	Succinate dehydrogenase 1–1 isoform 1	70.44/6.19	Oxidoredutase
	gi|508699218		
239	Peroxidase	39.48/5.64	Response to oxidative stress
	gi|508724908		
252	Pathogenesis-related	29.42/3.99	Response to biotic stimulus
	protein P2 isoform 1		
	gi|508719160		
	Glutathione S transferase	25.23/5.68 26.74/6.22	Response to stress
257 260	Tc01 g015120		
	Tc04 g022550		
259	Proteasome subunit alfa tipo 6	26.74/6.22 27.13/6.09	Proteolytic activity
	Tc09 g006750		
266	Isocitrate dehydrogenase V isoform 1	40.99/6.68	Stress response
	gi|508715960		
286 223	Elongation factor 1-gamma 3 isoform 1	48.39/5.95	Protein biosynthesis
	gi|508705116		
297	Cytosolic NADP+ dependent isocitrate dehydrogenase isoform 1 gi|508710350	52.46/8.86	Stress response
306	Regulatory particle triple-A ATPase 3 isoform 2	32.74/5.94	ATP synthesis
	gi|508727313		
335	Phosphoglucomutase/ phosphomannomutase family protein isoform 1	63.50/5.40	Carbohydrate metabolic process
	gi|508709149		
336	Vacuolar H+-ATPase	69.16/5.37	ATP synthesis
	gi|131573315		

**Table 3:** Protein expression in roots of CCN-10 x SCA-6 progeny identified by mass spectrometry.

Spot N°	Protein	MW(KDa)/pI	Biological process
56	Aspartic proteinase A1 isoform 1	60.49/5.04	Proteolysis Lipid metabolic process
	gi|508701345		
57 78	γ - Anidrase carbônica parcial	29.55/5.78	Carbon utilization
	Tc08 g002330	41.64/6.57	
83	PfkB-like carbohydrate	35.37/5.26	Kinase activity
	Kinase family protein		
	gi|508712952		
125	DC1 domain-containing	65.56/4.96	Metabolic process
	protein gi|508708602		
133 136	Protein mitochondrial outer membrane porin	15.83/8.20 29.56/8.64	Transport of ions and metabolites
	Tc00 g048560		
	Tc04 g008150		
134	Proteína serina / treonina fosfatase 2A da subunidade reguladora-β	50.03/9.12	Stimulate the activity of the fosfotirosina phosphatase
	Tc01 g016250		PP2A phosphotyrosine
142	Thaumatin-like	24.16/4.39	Response to stress
	Tc03 g026960		
224	Peroxidase	39.48/5.64	Response to oxidative stress
	gi|508724908		

## Discussion

### Pb caused alterations in the organelles and peroxidation of the nuclear membrane of cells of foliar and root mesophylls

Despite the low mobility in plants, Pb, in high concentrations, can easily reach the central cylinder and reach the shoots. Ultrastructural studies show that heavy metals accumulate in the cell wall, vacuoles and intercellular spaces [8, 24,38] and that small deposits are found in organelles such as mitochondria, nuclei and chloroplasts [4,53]. The deposition of Pb in cell walls is considered an efficient strategy of plants to gain tolerance to this metallic element [54]. The ultrastructural analyses of both evaluated progenies of *T*. *cacao*, from seeds treated with different concentrations of Pb in solution, presented electrodense deposits in the xylem cells of the roots and of the endoderm (Fig 3B and 3D), between the intracellular spaces and on the mesophyll cell wall, in the highest concentration of Pb (0.8 g L^-1^) (Figs 1D and 2D).

The increase in the amount of electrodense deposits, in parallel with the increased concentration of Pb, may indicate the presence of Pb in these deposits. Generally, most of the Pb is retained in the cell wall, due to the pectins which bind easily to some toxic metals such as Pb, promoting their immobilization [55]. The resistant progeny was more efficient in retaining Pb in the intercellular spaces, in the wall and on the inside of the endoderm cells, preventing that part of the Pb reach the central cylinder and be transported to shoots. However, the xylem cells of the susceptible progeny presented a high concentration of electrodense compounds, indicating that greater amounts of Pb reached the shoots of the seedlings of this progeny. In this same progeny, there was a considerable amount of plastoglobules in the chloroplasts of the foliar mesophyll cells, mainly for the highest dose of Pb (0.8 g L^-1^) (Fig 1F). The plastoglobules are structures that exist within the chloroplasts that are attached to the thylakoids [53], working as a reservoir of lipids and serving as an active site of synthesis and recycling of protein under stress conditions [56]. The number and size of plastoglobules can increase after exposure to heavy metal stress [50]. Studies reported the presence of plastoglobules in seedlings of *Solanum lycopersicom* subjected to high concentrations of Pb [57]. Therefore, the increase of these globules of lipoproteins, which were observed mainly in the susceptible progeny, can represent a mechanism used by this species to avoid possible damage to the photosynthetic apparatus.

Probably the damage evidenced in the chloroplasts and in the nucleus of the susceptible progeny was due to the fact that this progeny cannot immobilize Pb, with greater efficiency, in the roots (Fig 3B). In addition to the fact that the ultrastructural analyses demonstrated degradation of nuclear membrane and malformation of chloroplasts, the increased concentration of TBARS found in the leaves of this same progeny reflects the level of lipid peroxidation in the cellular membrane. The TBARS result from the lipid peroxidation that occurs in cell membranes of plant tissues, when they are exposed to different environmental stresses [58]. In the resistant progeny, Pb did not cause damage to cells of foliar mesophyll tissues, which may be verified by the small accumulation of TBARS and by the low activity of guaiacol peroxidase. However, in the roots of this same progeny, TBARS accumulation was significant in comparison to the control, even with the high activity of peroxidases. In contrast, in the susceptible progeny, the level of TBARS in roots was expressive in the highest concentrations of Pb, due to little activity of peroxidases in the cells of these tissues, mainly in the highest concentration of Pb.

### Pb increased guaiacol peroxidase activity

The exposure of plants to heavy metals such as Pb inevitably leads to the production of reactive oxygen species (ROSs), which when not metabolized cause serious damage to plnat cells and tissues. To tackle and repair the damage caused by ROSs, plants have developed complex systems of enzymatic and non-enzymatic antioxidants. Among the enzymatic antioxidants, in the present work, the guaiacol peroxidase activity (GPX) in leaves and roots of progenies of *T*. cacao, were analyzed. The activity of this enzyme in the leaves of the susceptible progeny increased with increasing doses of Pb via seminal (Fig 6A); contrary to what occurred with the resistant progeny, in which the major activity was observed only in the treatment of 0.2 g Pb L^-1^ (Fig 6A). This interprogenic difference may be due to the fact that resistant seedlings can retain greater amounts of Pb in roots. In contrast, there was an increase in the activity of GPX, parallel with the increase in doses of Pb, in the roots of both progenies, except for the highest dose of Pb (0.8 g L^-1^) for the susceptible progeny (Fig 6B). Similar results are found in several works [5,54,59–61].

The peroxidases act by decomposing the H_2_O_2_ from the dismutation of the superoxide radicals by the action of the superoxide dismutase (SOD) in molecular oxygen and water. The ability to maintain the activity of peroxidases in high levels, under conditions of environmental stress, is essential so that there is balance between the formation and the removal of H_2_O_2_ from the intracellular environment [62]. However, at the highest dose of Pb, in the susceptible progeny, GPX activity was low in roots, indicating a delay in the removal of H_2_O_2_ and, consequently, increase of lipid peroxidation of radicular tissues cell membranes. Similar results were also found by other authors [57,63], which proposed that the production of ROSs exceeded the capacity of removal, inducing oxidative stress. In addition, the increase in the Fe content, parallel to the increase in concentration of Pb in the roots of both progenies, may indicate that Fe is promoting detoxification of ROSs, as many works have demonstrated in plant roots of *Rhodes grass* [63] and in *Vallisneria natans* [19].

### Pb induced the expression of genes involved in the mechanism of enzymatic and non-enzymatic defense

Several genes are involved in cellular responses to various types of biotic and abiotic stresses. Through the variation in accumulation of mRNA of target genes, the level of relative expression of three genes was measured in the leaves and roots of the susceptible and resistant progeny. The PER and Cu-Zn SOD_Cyt_ genes, whose end products are enzymes that catalyze antioxidant reactions, presented higher expression in roots of both the evaluated progenies of *T*. *cacao* (Fig 7C–7F). In the resistant progeny, repression of the PER gene occurred in leaves and higher expression occurred in roots (Fig 7F). These results corroborate with those found in the analysis of GPX activity, for which the activity of this enzyme was similar to that of the control in the leaves, and had increased activity in roots (Fig 7A and 7B). However, in the susceptible progeny, the expression of PER was very low in the leaves and roots (Fig 7A), while the GPX activity was high in both organs of this progeny (Fig 7A and 7B). This could suggest that the expression occurred, however, at some point before the collection of the biological material, performed at 60 DAE. Furthermore, other peroxidases such as catalase and ascorbate peroxidase could have been acting in the removal of ROS. Recent studies have shown that Pb induce the expression of genes encoding the enzymes glutathione reductase, glutathione S-transferase and ascorbate peroxidase, antioxidant enzymes responsible for the plant defense against ROS [29,33,35]. In contrast, the expression of Cu-Zn SOD_Cyt_ was low in the leaves in both progenies (Fig 7C and 7D) and high in the roots, especially in the susceptible progeny. The increase of the activity of SOD and of other antioxidant enzymes is attributed to the increase in concentration of ROSs, and these, in turn, act as indicators of transcription in the induction of genes of biosynthesis of these enzymes [64].

Other important antioxidants that participate in the non-enzymatic defense mechanisms of plants under heavy metal stress, are the phytochelatin synthases (PCs), consisting of peptides synthesized enzymatically, whose biosynthesis is stimulated by the free metal concentration present in the cell, and uses Glutathione (GSH) as substrate [65,66]. Both are thiols of low molecular weight and have great affinity for heavy metals. The PCs form complexes with the toxic metal in the cytosol and, subsequently, transports it into the vacuole, detoxifying the cell [29,66]. The GSH also acts as an antioxidant agent, in addition to being the precursor of the synthesis of PCs [27].

Synthesis of PCs, in response to Pb, and formation of the complex PC-Pb were reported in works carried out with legumes [67,68], and also with other heavy metals such as Cd [69]. Cd induced a significant increase in the level of mRNA expression of genes involved in the synthesis of PCs in leaves of *A*. *thaliana*. In the progenies of *T*. *cacao* evaluated in this study, there was an increase in the expression of PCs in the roots of the susceptible progeny, for the highest dose of Pb applied via seminal (0.8 g L^-1^), and in the doses of 0.2 and 0.8 g Pb L^-1^ in roots of the resistant progeny (Fig 9A and 9B).

### Contents of Pb and macro and mineral micronutrients

The essential mineral elements, such as K, P, Ca, Mg, Mn, S, Cu, Zn and Fe, are important to the growth and development of plant species. These elements are involved in different biosynthetic pathways and are cofactors of several enzymes [70]. The toxicity caused by Pb altered the uptake and translocation of mineral nutrients in the progenies of CCN-10 x SCA-6 and *Catongo*. Studies show that the Pb competes with other essential mineral elements that are transported in plants [19,71,72]. The observed decrease in the concentrations of K in Catongo and of Cu in the leaves and stems of CCN-10 x SCV-6 can be assigned to competition by Pb (Figs 9F and 10A). Similar results were found in *Brassica oleracea* [62] and *Vallisneria natans* [19]. The significant Pb (p< 0.05) promoted an increase of K in the stem of the progeny of CCN-10 x SCA-6 (Fig 9E). Reported results unlike the plants by other species exposed to Pb [62,73]. How K participates in the activation of several enzymes, probably the increased concentration of the K favored the tolerance of this progeny to Pb.

### Pb induced the expression of proteins related to oxidative stress

Proteomic analysis by means of two-dimensional SDS-PAGE has been an effective tool for differentiating the protein profile of various genotypes of a same species when subjected to the same stress. Among the 24 spots identified in roots of the susceptible progeny, spots 210, 239, 257, and 260 are enzymes that act as antioxidants, responsible for maintaining the production of ROSs under control, avoiding the toxic effects of agents such as Pb [39,74]. APXs and GST, in addition to acting on the antioxidative metabolism in the removal of ROSs, these enzymes participate in the glutathione-ascorbate cycle (ASA-GSH) [75]. In this process, ascorbate is used as a substrate in the synthesis of glutathione, which is composed of a group of multifunctional enzymes, and catalyzes the conjugation of glutathione (GSH) with heavy metals, which are stored in the vacuole for cellular detoxification in plants [27,76].

*Macrotyloma uniflorum* and *Cicer arietinum* plants, subjected to different concentrations of Pb, presented increased activity of GST [70]. The cycle of ASA-GSH triggers a series of reactions involving important enzymes and metabolites with redox properties for the efficient elimination of most ROSs produced at the cellular level [28,77]. Pb induced up-regulation of other proteins, among which spots 197, 202, 229, 297, and 266, are highlighted. ALDH, spot 229, has played an important role in the detoxification of aldehydes generated by oxidative stress in plants. The super-expression of these enzymes has been performed in transgenic plants of *Arabidopsis* exposed to heavy metals [78]. The cytosolic NADP±-dependent isocitrate dehydrogenase (IDPC) detected in the susceptible progeny, spots 297 and 266, has the function of catalyzing the oxidative decarboxylation of isocitrate to oxo-glutarate and requires NAD^+^ or NADP^+^. Studies conducted with the form (IDPC), using transformed cells, showed their importance against oxidative damage since IDPC is responsible for the production of NADPH, which is used in the recycling of GSH and removal of H_2_O_2_ [79]. This protein belongs to the multigenic family PR-5, and its expression is induced by various biotic and abiotic stresses [80]. The expression of the osmotin protein, spot 197, in the susceptible progeny may be associated to a possible water shortage in seedlings, due to the rupture and death of cells of the radicular system promoted by Pb. Some studies reported a decline in the rate of transpiration and relative water content in plants that grow under exposure to Pb [81], since Pb decreases the level of compounds that are associated with the maintenance of cell turgor pressure and with cell wall plasticity and, therefore, reduces the water potential inside the cell [4]. Another protein that has been very important to plants, when subjected to metals such as Zn, Hg, and Cu is the aspartic protein [28,82], since some metals stimulate its hydrolytic activity. Aspartic protein was detected in both progenies of *T*. *cacao* evaluated, spots 202 and 56 (Fig 11B and 11D). In the control seedlings of these progenies, these enzymes were absent or undetectable. In the resistant progeny, only one antioxidant enzyme was detected, spot 224. However, other proteins such as thaumatin-like, spot 142, and aspartic protein were detected. Studies have shown that thaumatin-like is induced as a result of H_2_O_2_ production in plants, caused by biotic and abiotic stress, such as increase in salinity, drought, and heavy metal concentration [38,83,84].

The expression of proteins involved in specific pathways of detoxification of metals or in the protection and repair of metabolic pathways is important to mitigate the damage caused to plants by the presence of metals. However, plants present different responses when subjected to the same stress. This difference is attributed, mainly, to the genotypic constitution of each species. The progeny *Catongo*, considered susceptible to biotic stress, was less tolerant to the stress caused by Pb. In this progeny, the presence of Pb induced the expression of several enzymes related to oxidative stress, indicating that Pb caused damage to the cellular compartments, generating free radicals, which, in turn, induced oxidative stress. In CCN-10 x SCA-6, Pb did not cause enough damage to induce oxidative stress. This progeny presents greater heterozygosity, since it is the result of the cross between two genotypes considered highly tolerant, featuring, therefore, a variety of genes which contribute to enhance resistance to the plant.

## Supporting Information

S1 Table Concentration of thiobarbituric acid reactive substances (TBARS). Leaves and roots of two progenies of *T*. *cacao* exposed to increasing doses of Pb. Mean values intraprogenies followed by the same lowercase letters do not differ by Tukey test (p<0.05). Mean values of four replicates (± SE).
(XLSX)

S2 Table Guaiacol peroxidase activity (POD). Leaves and roots of two progenies of *T*. *cacao* exposed to increasing doses of Pb. The statistical significance interprogenies was obtained by t-test. (*) p<0.05; (**) p<0.01; (***) p<0.001; (ns) not significant. Mean values intraprogenies followed by the same lowercase letters do not differ by Tukey test (p<0.05). Mean values of four replicates (± SE).
(XLSX)

S3 Table Amount of gene transcripts of the biosynthetic pathway of cytoplasmic superoxide dismutase (SODCyt) in leaves and roots of *T*. *cacao* progenies. Catongo and CCN-10 x SCA-6 exposed to increasing of Pb doses. The mRNA levels were quantified by quantitative real-time PCR. The mRNA levels were normalized in respect to tubulin, and are expressed relatively to those of control plants that were given a value of 1. Mean values of Intraprogenies followed by the same lowercase letters do not differ by Tukey test (p <0.05). Mean values of six replicates (± SE).
(XLSX)

S4 Table Amount of gene transcripts of the biosynthetic pathway of peroxidase (PER-1) in leaves and roots of *T*. *cacao* progenies. Catongo and CCN-10 x SCA-6 exposed to increasing of Pb doses. The mRNA levels were quantified by quantitative real-time PCR. The mRNA levels were normalized in respect to tubulin, and are expressed relatively to those of control plants that were given a value of 1. Mean values of Intraprogenies followed by the same lowercase letters do not differ by Tukey test (p <0.05). Mean values of six replicates (± SE).
(XLSX)

S5 Table Amount of gene transcripts of the biosynthetic pathway of phytochelatins synthase (PCs). Catongo and CCN-10 x SCA-6 exposed to increasing of Pb doses. The mRNA levels were quantified by quantitative real-time PCR. The mRNA levels were normalized in respect to tubulin, and are expressed relatively to those of control plants that were given a value of 1. Mean values of Intraprogenies followed by the same lowercase letters do not differ by Tukey test (p <0.05). Mean values of six replicates (± SE).
(XLSX)

S6 Table Accumulation of mineral micronutrients (Cu, Fe, Mn, Zn and Pb) in roots, stem and leaves of two progenies of T. cacao exposed to increasing Pb doses. CCN-10 x SCA-6 and Catongo.
(XLSX)

S7 Table Accumulation of mineral macronutrients (Mg, Ca, K) in roots, stem and leaves of two progenies of T. cacao exposed to increasing of Pb doses. CCN-10 x SCA-6 and Catongo.
(XLSX)
